# 4-Diphenyl­phosphanyl-1,5-naphthyridine

**DOI:** 10.1107/S1600536812039992

**Published:** 2012-09-26

**Authors:** Ya-Ming Wu

**Affiliations:** a Department of Applied Chemistry, Nanjing College of Chemical Technology, No. 625 Geguan Road, Dachang, Nanjing 210048, People’s Republic of China

## Abstract

The asymmetric unit of the title compound, C_20_H_15_N_2_P, contains two independent mol­ecules with similar structures. The 1,5-naphthyridine ring system is nearly planar, with maximum deviations of 0.010 (3) and 0.012 (3) Å; its mean plane is oriented with respect to the two phenyl rings at 79.69 (12) and 84.00 (10)° in one mol­ecule, and at 74.25 (12) and 82.05 (11)° in the other. The two phenyl rings are twisted with respect to each other with a dihedral angle of 75.96 (14)° in one mol­ecule and 86.30 (13)° in the other.

## Related literature

For applications of the title compound, see: Badawneh *et al.* (2001 ▶); Hawes *et al.* (1977 ▶); Goswami & Mukherjee (1997 ▶); Goswami *et al.* (2001 ▶, 2005 ▶). For the synthesis of the title compound, see: Chen *et al.* (2012 ▶).
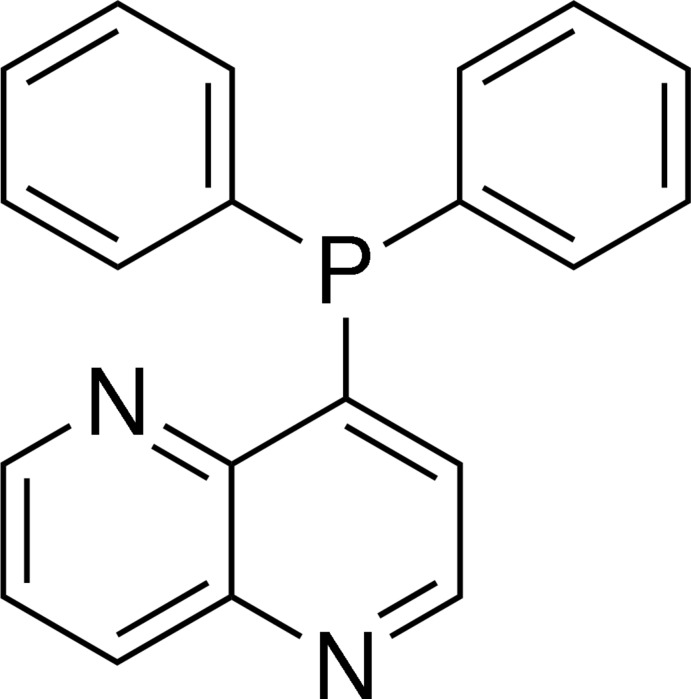


## Experimental

### 

#### Crystal data

C_20_H_15_N_2_P
*M* *_r_* = 314.31
Triclinic, 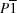

*a* = 10.1103 (7) Å
*b* = 11.7020 (8) Å
*c* = 15.7060 (11) Å
α = 71.54 (3)°
β = 75.05 (3)°
γ = 71.37 (3)°
*V* = 1644.6 (4) Å^3^
*Z* = 4
Mo *K*α radiation
μ = 0.17 mm^−1^
*T* = 293 K
0.30 × 0.20 × 0.20 mm

#### Data collection

Enraf–Nonius CAD-4 diffractometer
Absorption correction: ψ scan (North *et al.*, 1968 ▶) *T*_min_ = 0.952, *T*_max_ = 0.967
6393 measured reflections
6024 independent reflections
4473 reflections with *I* > 2σ(*I*)
*R*_int_ = 0.019
3 standard reflections every 200 reflections intensity decay: 1%

#### Refinement

*R*[*F* ^2^ > 2σ(*F* ^2^)] = 0.047
*wR*(*F* ^2^) = 0.151
*S* = 1.00
6024 reflections
416 parameters
H-atom parameters constrained
Δρ_max_ = 0.24 e Å^−3^
Δρ_min_ = −0.18 e Å^−3^

### 

Data collection: *CAD-4 EXPRESS* (Enraf–Nonius, 1994 ▶); cell refinement: *CAD-4 EXPRESS*; data reduction: *XCAD4* (Harms & Wocadlo, 1995 ▶); program(s) used to solve structure: *SHELXS97* (Sheldrick, 2008 ▶); program(s) used to refine structure: *SHELXL97* (Sheldrick, 2008 ▶); molecular graphics: *SHELXTL* (Sheldrick, 2008 ▶); software used to prepare material for publication: *SHELXTL*.

## Supplementary Material

Crystal structure: contains datablock(s) I, global. DOI: 10.1107/S1600536812039992/xu5619sup1.cif

Structure factors: contains datablock(s) I. DOI: 10.1107/S1600536812039992/xu5619Isup2.hkl

Supplementary material file. DOI: 10.1107/S1600536812039992/xu5619Isup3.cml

Additional supplementary materials:  crystallographic information; 3D view; checkCIF report

## Figures and Tables

**Fig. 1.**
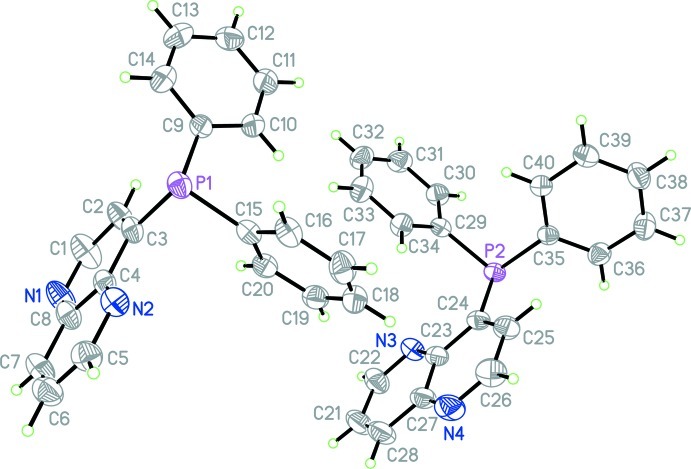
The molecular structure of (I), with the atom-numbering scheme. Displacement ellipsoids are drawn at the 50% probability level.

**Fig. 2.**
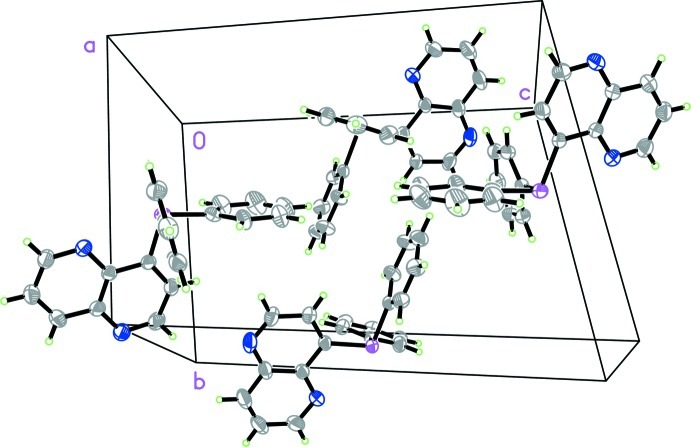
A packing diagram of (I).

## supplementary crystallographic information

### Comment

The title compound, (I), is an important intermediate in medicine (Badawneh *et al.*, 2001; Hawes *et al.*, 1977). Naphthyridines are also used as a key molecule in molecular recognition chemistry (Goswami & Mukherjee, 1997; Goswami *et al.*, 2005, 2001).

The molecular structure of (I) is shown in Fig. 1. The asymmetric unit of the title compound, C_20_H_15_N_2_P, contains two independent molecules with the similar structure. The 1,5-naphthrydine ring system is nearly planar with the maximum deviation of 0.010 (3) and 0.012 (3) Å, respectively; its mean plane is oriented with respect to the two phenyl rings at 79.69 (12) and 84.00 (10)° in one molecule and 74.25 (12) and 82.05 (11)° in the other. The two phenyl rings are twisted to each other with a dihedral angle of 75.96 (14)° in one molecule and 86.30 (13)° in the other.

The crystal packing of the molecules in the crystal is influenced by van der Waals forces.

### Experimental

The title compound was synthesized according to the published procedure (Chen *et al.*, 2012). Crystals suitable for X-ray analysis were obtained by dissolving it(0.5 g) in tetrahydrofuran (20 ml) and evaporating the solvent slowly at room temperature for about 5 d.

### Refinement

H atoms were positioned geometrically, with C—H = 0.93 Å for aromatic, and constrained to ride on their parent atoms, with *U*ĩso~(H) = *xU*~eq~(C), where *x* = 1.2 for aromatic H,and *x* = 1.5 for other H.

### Crystal data

**Table:**

C_20_H_15_N_2_P	*Z* = 4
*M**_r_* = 314.31	*F*(000) = 656
Triclinic, *P*1	*D*_x_ = 1.269 Mg m^−^^3^
Hall symbol: -P 1	Mo *K*α radiation, λ = 0.71073 Å
*a* = 10.1103 (7) Å	Cell parameters from 25 reflections
*b* = 11.7020 (8) Å	θ = 10–14°
*c* = 15.7060 (11) Å	µ = 0.17 mm^−^^1^
α = 71.54 (3)°	*T* = 293 K
β = 75.05 (3)°	Block, yellow
γ = 71.37 (3)°	0.30 × 0.20 × 0.20 mm
*V* = 1644.6 (4) Å^3^	

### Data collection

**Table:**

Enraf–Nonius CAD-4 diffractometer	4473 reflections with *I* > 2σ(*I*)
Radiation source: fine-focus sealed tube	*R*_int_ = 0.019
Graphite monochromator	θ_max_ = 25.4°, θ_min_ = 1.4°
ω/2θ scans	*h* = 0→12
Absorption correction: ψ scan (North *et al.*, 1968)	*k* = −13→14
*T*_min_ = 0.952, *T*_max_ = 0.967	*l* = −18→18
6393 measured reflections	3 standard reflections every 200 reflections
6024 independent reflections	intensity decay: 1%

### Refinement

**Table:**

Refinement on *F*^2^	Secondary atom site location: difference Fourier map
Least-squares matrix: full	Hydrogen site location: inferred from neighbouring sites
*R*[*F*^2^ > 2σ(*F*^2^)] = 0.047	H-atom parameters constrained
*wR*(*F*^2^) = 0.151	*w* = 1/[σ^2^(*F*_o_^2^) + (0.1*P*)^2^ + 0.1*P*] where *P* = (*F*_o_^2^ + 2*F*_c_^2^)/3
*S* = 1.00	(Δ/σ)_max_ < 0.001
6024 reflections	Δρ_max_ = 0.24 e Å^−^^3^
416 parameters	Δρ_min_ = −0.18 e Å^−^^3^
0 restraints	Extinction correction: *SHELXL97* (Sheldrick, 2008), Fc^*^=kFc[1+0.001xFc^2^λ^3^/sin(2θ)]^-1/4^
Primary atom site location: structure-invariant direct methods	Extinction coefficient: 0.056 (4)

### Special details

**Table:**

Geometry. All e.s.d.'s (except the e.s.d. in the dihedral angle between two l.s. planes) are estimated using the full covariance matrix. The cell e.s.d.'s are taken into account individually in the estimation of e.s.d.'s in distances, angles and torsion angles; correlations between e.s.d.'s in cell parameters are only used when they are defined by crystal symmetry. An approximate (isotropic) treatment of cell e.s.d.'s is used for estimating e.s.d.'s involving l.s. planes.
Refinement. Refinement of *F*^2^ against ALL reflections. The weighted *R*-factor *wR* and goodness of fit *S* are based on *F*^2^, conventional *R*-factors *R* are based on *F*, with *F* set to zero for negative *F*^2^. The threshold expression of *F*^2^ > σ(*F*^2^) is used only for calculating *R*-factors(gt) *etc*. and is not relevant to the choice of reflections for refinement. *R*-factors based on *F*^2^ are statistically about twice as large as those based on *F*, and *R*- factors based on ALL data will be even larger.

### Fractional atomic coordinates and isotropic or equivalent isotropic displacement parameters (Å^2^)

**Table:**

	*x*	*y*	*z*	*U*_iso_*/*U*_eq_	
P1	0.66814 (7)	0.57637 (5)	0.05824 (4)	0.0532 (2)	
N1	0.4162 (3)	0.9483 (2)	−0.11519 (15)	0.0820 (7)	
C1	0.3612 (3)	0.8979 (3)	−0.0324 (2)	0.0875 (10)	
H1B	0.2700	0.9387	−0.0093	0.105*	
N2	0.7623 (2)	0.71544 (18)	−0.12779 (13)	0.0590 (5)	
C2	0.4291 (3)	0.7868 (3)	0.02464 (17)	0.0673 (7)	
H2B	0.3821	0.7559	0.0827	0.081*	
C3	0.5643 (2)	0.7238 (2)	−0.00517 (14)	0.0504 (5)	
C4	0.6276 (2)	0.7760 (2)	−0.09501 (15)	0.0499 (5)	
C5	0.8181 (3)	0.7656 (3)	−0.21080 (18)	0.0682 (7)	
H5A	0.9100	0.7258	−0.2335	0.082*	
C6	0.7487 (3)	0.8749 (3)	−0.26760 (19)	0.0747 (8)	
H6A	0.7934	0.9061	−0.3263	0.090*	
C7	0.6156 (3)	0.9346 (3)	−0.23573 (18)	0.0760 (8)	
H7A	0.5676	1.0073	−0.2726	0.091*	
C8	0.5500 (3)	0.8869 (2)	−0.14705 (16)	0.0604 (6)	
C9	0.5452 (3)	0.5467 (2)	0.16628 (16)	0.0530 (6)	
C10	0.5550 (3)	0.5700 (2)	0.24469 (16)	0.0638 (7)	
H10A	0.6229	0.6092	0.2429	0.077*	
C11	0.4666 (3)	0.5367 (3)	0.32552 (19)	0.0778 (8)	
H11A	0.4764	0.5521	0.3778	0.093*	
C12	0.3649 (3)	0.4813 (3)	0.3295 (2)	0.0857 (9)	
H12A	0.3045	0.4599	0.3843	0.103*	
C13	0.3518 (4)	0.4573 (3)	0.2530 (3)	0.0998 (11)	
H13A	0.2826	0.4192	0.2554	0.120*	
C14	0.4404 (4)	0.4896 (3)	0.1730 (2)	0.0858 (9)	
H14A	0.4304	0.4728	0.1212	0.103*	
C15	0.7959 (2)	0.6320 (2)	0.08854 (14)	0.0490 (5)	
C16	0.9299 (3)	0.5519 (2)	0.09739 (16)	0.0586 (6)	
H16A	0.9516	0.4729	0.0880	0.070*	
C17	1.0306 (3)	0.5877 (3)	0.11983 (18)	0.0705 (7)	
H17A	1.1205	0.5339	0.1239	0.085*	
C18	0.9986 (3)	0.7034 (3)	0.13635 (18)	0.0706 (7)	
H18A	1.0663	0.7271	0.1525	0.085*	
C19	0.8658 (3)	0.7839 (2)	0.12884 (16)	0.0613 (6)	
H19A	0.8439	0.8618	0.1403	0.074*	
C20	0.7658 (3)	0.7491 (2)	0.10448 (15)	0.0533 (6)	
H20A	0.6771	0.8043	0.0986	0.064*	
P2	0.87225 (6)	0.89905 (5)	0.46590 (4)	0.04682 (19)	
N3	0.8062 (2)	1.09211 (19)	0.29639 (13)	0.0635 (5)	
N4	1.1480 (3)	0.9364 (2)	0.17523 (15)	0.0769 (7)	
C21	0.8431 (4)	1.2010 (3)	0.1386 (2)	0.0902 (10)	
H21A	0.8080	1.2676	0.0920	0.108*	
C22	0.7647 (4)	1.1837 (3)	0.2274 (2)	0.0790 (8)	
H22A	0.6779	1.2409	0.2378	0.095*	
C23	0.9337 (3)	1.0089 (2)	0.27942 (15)	0.0533 (6)	
C24	0.9814 (2)	0.9077 (2)	0.35274 (15)	0.0500 (5)	
C25	1.1114 (3)	0.8266 (2)	0.33337 (17)	0.0612 (6)	
H25A	1.1475	0.7590	0.3791	0.073*	
C26	1.1890 (3)	0.8465 (3)	0.24434 (19)	0.0763 (8)	
H26A	1.2769	0.7907	0.2340	0.092*	
C27	1.0201 (3)	1.0188 (3)	0.19230 (16)	0.0646 (7)	
C28	0.9701 (4)	1.1202 (3)	0.12131 (19)	0.0832 (9)	
H28A	1.0240	1.1315	0.0630	0.100*	
C29	0.7225 (2)	0.85058 (19)	0.45554 (14)	0.0471 (5)	
C30	0.6058 (3)	0.8561 (2)	0.52547 (16)	0.0610 (6)	
H30A	0.6070	0.8830	0.5749	0.073*	
C31	0.4878 (3)	0.8223 (3)	0.5231 (2)	0.0713 (7)	
H31A	0.4111	0.8260	0.5708	0.086*	
C32	0.4836 (3)	0.7835 (3)	0.4509 (2)	0.0721 (7)	
H32A	0.4041	0.7613	0.4490	0.087*	
C33	0.5972 (3)	0.7775 (3)	0.3813 (2)	0.0725 (7)	
H33A	0.5944	0.7509	0.3320	0.087*	
C34	0.7168 (3)	0.8104 (2)	0.38273 (16)	0.0581 (6)	
H34A	0.7932	0.8056	0.3348	0.070*	
C35	0.9758 (2)	0.7539 (2)	0.53228 (14)	0.0457 (5)	
C36	1.0835 (3)	0.7605 (2)	0.56829 (17)	0.0596 (6)	
H36A	1.1033	0.8369	0.5566	0.072*	
C37	1.1621 (3)	0.6555 (3)	0.62125 (19)	0.0711 (7)	
H37A	1.2359	0.6611	0.6437	0.085*	
C38	1.1314 (3)	0.5431 (3)	0.64080 (19)	0.0710 (7)	
H38A	1.1833	0.4725	0.6773	0.085*	
C39	1.0240 (3)	0.5347 (2)	0.6064 (2)	0.0745 (8)	
H39A	1.0029	0.4584	0.6200	0.089*	
C40	0.9475 (3)	0.6386 (2)	0.55198 (17)	0.0614 (6)	
H40A	0.8761	0.6318	0.5281	0.074*	

### Atomic displacement parameters (Å^2^)

**Table:**

	*U*^11^	*U*^22^	*U*^33^	*U*^12^	*U*^13^	*U*^23^
P1	0.0635 (4)	0.0426 (3)	0.0511 (4)	−0.0040 (3)	−0.0171 (3)	−0.0128 (3)
N1	0.0852 (17)	0.0746 (15)	0.0546 (13)	0.0178 (13)	−0.0169 (12)	−0.0088 (11)
C1	0.0704 (18)	0.090 (2)	0.0652 (18)	0.0263 (16)	−0.0094 (14)	−0.0193 (16)
N2	0.0597 (12)	0.0575 (12)	0.0549 (12)	−0.0071 (10)	−0.0067 (9)	−0.0187 (10)
C2	0.0653 (16)	0.0725 (16)	0.0443 (13)	0.0034 (13)	−0.0069 (11)	−0.0116 (12)
C3	0.0597 (14)	0.0474 (12)	0.0426 (12)	−0.0036 (10)	−0.0150 (10)	−0.0149 (10)
C4	0.0605 (14)	0.0460 (12)	0.0444 (12)	−0.0068 (10)	−0.0135 (10)	−0.0167 (10)
C5	0.0675 (16)	0.0739 (17)	0.0613 (16)	−0.0179 (14)	0.0010 (13)	−0.0250 (14)
C6	0.095 (2)	0.0746 (18)	0.0524 (15)	−0.0277 (16)	−0.0019 (14)	−0.0165 (14)
C7	0.101 (2)	0.0638 (16)	0.0503 (15)	−0.0100 (16)	−0.0188 (15)	−0.0035 (13)
C8	0.0704 (16)	0.0541 (14)	0.0494 (13)	−0.0017 (12)	−0.0138 (12)	−0.0155 (11)
C9	0.0602 (14)	0.0435 (12)	0.0547 (13)	−0.0128 (10)	−0.0205 (11)	−0.0043 (10)
C10	0.0676 (16)	0.0775 (17)	0.0527 (14)	−0.0356 (14)	−0.0129 (12)	−0.0065 (12)
C11	0.0804 (19)	0.100 (2)	0.0571 (16)	−0.0427 (17)	−0.0135 (14)	−0.0053 (15)
C12	0.083 (2)	0.094 (2)	0.0741 (19)	−0.0470 (18)	−0.0125 (16)	0.0091 (17)
C13	0.108 (3)	0.112 (3)	0.101 (3)	−0.075 (2)	−0.023 (2)	−0.007 (2)
C14	0.106 (2)	0.096 (2)	0.078 (2)	−0.056 (2)	−0.0270 (18)	−0.0154 (17)
C15	0.0540 (13)	0.0454 (12)	0.0397 (11)	−0.0062 (10)	−0.0063 (9)	−0.0083 (9)
C16	0.0592 (14)	0.0547 (14)	0.0522 (13)	0.0019 (11)	−0.0110 (11)	−0.0164 (11)
C17	0.0540 (15)	0.0788 (19)	0.0704 (17)	0.0001 (13)	−0.0183 (13)	−0.0195 (14)
C18	0.0655 (17)	0.087 (2)	0.0628 (16)	−0.0275 (15)	−0.0177 (13)	−0.0104 (14)
C19	0.0762 (17)	0.0544 (14)	0.0546 (14)	−0.0234 (13)	−0.0091 (12)	−0.0110 (11)
C20	0.0538 (13)	0.0449 (12)	0.0535 (13)	−0.0060 (10)	−0.0081 (10)	−0.0099 (10)
P2	0.0514 (3)	0.0457 (3)	0.0399 (3)	−0.0145 (3)	−0.0011 (2)	−0.0099 (2)
N3	0.0741 (14)	0.0552 (12)	0.0528 (12)	−0.0134 (11)	−0.0118 (10)	−0.0054 (10)
N4	0.0797 (16)	0.0955 (18)	0.0497 (13)	−0.0305 (14)	0.0115 (11)	−0.0212 (13)
C21	0.131 (3)	0.076 (2)	0.0599 (18)	−0.040 (2)	−0.0297 (19)	0.0105 (15)
C22	0.100 (2)	0.0591 (16)	0.0706 (18)	−0.0137 (15)	−0.0275 (16)	−0.0041 (14)
C23	0.0656 (15)	0.0512 (13)	0.0450 (12)	−0.0242 (11)	−0.0039 (11)	−0.0102 (10)
C24	0.0541 (13)	0.0511 (12)	0.0437 (12)	−0.0201 (10)	0.0004 (10)	−0.0113 (10)
C25	0.0613 (15)	0.0650 (15)	0.0517 (14)	−0.0169 (12)	0.0016 (11)	−0.0161 (12)
C26	0.0690 (17)	0.091 (2)	0.0613 (17)	−0.0173 (15)	0.0115 (14)	−0.0318 (16)
C27	0.0821 (19)	0.0720 (16)	0.0451 (13)	−0.0389 (15)	−0.0019 (12)	−0.0106 (12)
C28	0.113 (3)	0.089 (2)	0.0459 (15)	−0.046 (2)	−0.0079 (16)	0.0005 (15)
C29	0.0485 (12)	0.0437 (11)	0.0423 (11)	−0.0096 (9)	−0.0046 (9)	−0.0066 (9)
C30	0.0565 (14)	0.0710 (16)	0.0505 (13)	−0.0174 (12)	0.0016 (11)	−0.0166 (12)
C31	0.0525 (15)	0.0784 (18)	0.0716 (17)	−0.0200 (13)	0.0041 (13)	−0.0130 (15)
C32	0.0509 (15)	0.0708 (17)	0.093 (2)	−0.0185 (13)	−0.0168 (14)	−0.0125 (16)
C33	0.0710 (18)	0.0775 (18)	0.0796 (19)	−0.0197 (14)	−0.0203 (15)	−0.0284 (15)
C34	0.0558 (14)	0.0643 (15)	0.0550 (14)	−0.0151 (12)	−0.0057 (11)	−0.0199 (12)
C35	0.0473 (12)	0.0491 (12)	0.0387 (11)	−0.0152 (10)	−0.0005 (9)	−0.0112 (9)
C36	0.0610 (15)	0.0573 (14)	0.0650 (15)	−0.0188 (12)	−0.0118 (12)	−0.0178 (12)
C37	0.0668 (16)	0.0730 (18)	0.0808 (19)	−0.0118 (14)	−0.0299 (14)	−0.0225 (15)
C38	0.0730 (18)	0.0622 (16)	0.0714 (17)	−0.0063 (13)	−0.0283 (14)	−0.0071 (13)
C39	0.0841 (19)	0.0503 (14)	0.088 (2)	−0.0216 (14)	−0.0300 (16)	0.0002 (13)
C40	0.0645 (15)	0.0556 (14)	0.0657 (15)	−0.0242 (12)	−0.0207 (12)	−0.0015 (12)

### Geometric parameters (Å, º)

**Table:**

P1—C9	1.833 (2)	P2—C24	1.825 (2)
P1—C3	1.834 (2)	P2—C29	1.837 (2)
P1—C15	1.836 (2)	P2—C35	1.837 (2)
N1—C1	1.303 (3)	N3—C22	1.308 (3)
N1—C8	1.362 (3)	N3—C23	1.366 (3)
C1—C2	1.405 (4)	N4—C26	1.299 (4)
C1—H1B	0.9300	N4—C27	1.365 (4)
N2—C5	1.308 (3)	C21—C28	1.350 (5)
N2—C4	1.369 (3)	C21—C22	1.402 (4)
C2—C3	1.370 (3)	C21—H21A	0.9300
C2—H2B	0.9300	C22—H22A	0.9300
C3—C4	1.418 (3)	C23—C27	1.413 (3)
C4—C8	1.406 (3)	C23—C24	1.421 (3)
C5—C6	1.397 (4)	C24—C25	1.379 (3)
C5—H5A	0.9300	C25—C26	1.403 (3)
C6—C7	1.350 (4)	C25—H25A	0.9300
C6—H6A	0.9300	C26—H26A	0.9300
C7—C8	1.400 (4)	C27—C28	1.406 (4)
C7—H7A	0.9300	C28—H28A	0.9300
C9—C10	1.376 (3)	C29—C34	1.387 (3)
C9—C14	1.390 (4)	C29—C30	1.390 (3)
C10—C11	1.373 (3)	C30—C31	1.384 (4)
C10—H10A	0.9300	C30—H30A	0.9300
C11—C12	1.359 (4)	C31—C32	1.362 (4)
C11—H11A	0.9300	C31—H31A	0.9300
C12—C13	1.365 (5)	C32—C33	1.368 (4)
C12—H12A	0.9300	C32—H32A	0.9300
C13—C14	1.363 (4)	C33—C34	1.389 (4)
C13—H13A	0.9300	C33—H33A	0.9300
C14—H14A	0.9300	C34—H34A	0.9300
C15—C16	1.392 (3)	C35—C36	1.382 (3)
C15—C20	1.394 (3)	C35—C40	1.390 (3)
C16—C17	1.375 (4)	C36—C37	1.379 (3)
C16—H16A	0.9300	C36—H36A	0.9300
C17—C18	1.380 (4)	C37—C38	1.371 (4)
C17—H17A	0.9300	C37—H37A	0.9300
C18—C19	1.381 (4)	C38—C39	1.373 (4)
C18—H18A	0.9300	C38—H38A	0.9300
C19—C20	1.377 (3)	C39—C40	1.375 (3)
C19—H19A	0.9300	C39—H39A	0.9300
C20—H20A	0.9300	C40—H40A	0.9300
			
C9—P1—C3	101.25 (10)	C24—P2—C29	102.48 (10)
C9—P1—C15	102.27 (10)	C24—P2—C35	100.79 (10)
C3—P1—C15	100.59 (10)	C29—P2—C35	102.15 (9)
C1—N1—C8	116.2 (2)	C22—N3—C23	117.0 (2)
N1—C1—C2	125.0 (3)	C26—N4—C27	116.4 (2)
N1—C1—H1B	117.5	C28—C21—C22	119.5 (3)
C2—C1—H1B	117.5	C28—C21—H21A	120.3
C5—N2—C4	117.1 (2)	C22—C21—H21A	120.3
C3—C2—C1	120.1 (2)	N3—C22—C21	124.0 (3)
C3—C2—H2B	120.0	N3—C22—H22A	118.0
C1—C2—H2B	120.0	C21—C22—H22A	118.0
C2—C3—C4	116.5 (2)	N3—C23—C27	123.0 (2)
C2—C3—P1	126.31 (18)	N3—C23—C24	118.4 (2)
C4—C3—P1	117.19 (17)	C27—C23—C24	118.6 (2)
N2—C4—C8	122.6 (2)	C25—C24—C23	116.8 (2)
N2—C4—C3	118.4 (2)	C25—C24—P2	124.86 (18)
C8—C4—C3	119.1 (2)	C23—C24—P2	118.21 (17)
N2—C5—C6	124.3 (3)	C24—C25—C26	119.7 (2)
N2—C5—H5A	117.9	C24—C25—H25A	120.2
C6—C5—H5A	117.9	C26—C25—H25A	120.2
C7—C6—C5	118.8 (2)	N4—C26—C25	125.3 (3)
C7—C6—H6A	120.6	N4—C26—H26A	117.3
C5—C6—H6A	120.6	C25—C26—H26A	117.3
C6—C7—C8	119.9 (3)	N4—C27—C28	119.7 (3)
C6—C7—H7A	120.1	N4—C27—C23	123.1 (2)
C8—C7—H7A	120.1	C28—C27—C23	117.2 (3)
N1—C8—C7	119.5 (2)	C21—C28—C27	119.4 (3)
N1—C8—C4	123.2 (2)	C21—C28—H28A	120.3
C7—C8—C4	117.3 (2)	C27—C28—H28A	120.3
C10—C9—C14	116.6 (2)	C34—C29—C30	117.7 (2)
C10—C9—P1	124.08 (18)	C34—C29—P2	124.86 (17)
C14—C9—P1	119.2 (2)	C30—C29—P2	117.40 (17)
C11—C10—C9	121.4 (2)	C31—C30—C29	121.4 (2)
C11—C10—H10A	119.3	C31—C30—H30A	119.3
C9—C10—H10A	119.3	C29—C30—H30A	119.3
C12—C11—C10	120.4 (3)	C32—C31—C30	120.2 (2)
C12—C11—H11A	119.8	C32—C31—H31A	119.9
C10—C11—H11A	119.8	C30—C31—H31A	119.9
C11—C12—C13	119.7 (3)	C31—C32—C33	119.4 (2)
C11—C12—H12A	120.1	C31—C32—H32A	120.3
C13—C12—H12A	120.1	C33—C32—H32A	120.3
C14—C13—C12	119.8 (3)	C32—C33—C34	121.2 (3)
C14—C13—H13A	120.1	C32—C33—H33A	119.4
C12—C13—H13A	120.1	C34—C33—H33A	119.4
C13—C14—C9	122.1 (3)	C29—C34—C33	120.1 (2)
C13—C14—H14A	119.0	C29—C34—H34A	119.9
C9—C14—H14A	119.0	C33—C34—H34A	119.9
C16—C15—C20	118.2 (2)	C36—C35—C40	118.1 (2)
C16—C15—P1	118.02 (17)	C36—C35—P2	118.21 (17)
C20—C15—P1	123.79 (17)	C40—C35—P2	123.60 (17)
C17—C16—C15	121.0 (2)	C37—C36—C35	121.1 (2)
C17—C16—H16A	119.5	C37—C36—H36A	119.5
C15—C16—H16A	119.5	C35—C36—H36A	119.5
C16—C17—C18	120.1 (2)	C38—C37—C36	119.9 (2)
C16—C17—H17A	120.0	C38—C37—H37A	120.0
C18—C17—H17A	120.0	C36—C37—H37A	120.0
C17—C18—C19	119.9 (2)	C37—C38—C39	120.0 (2)
C17—C18—H18A	120.1	C37—C38—H38A	120.0
C19—C18—H18A	120.1	C39—C38—H38A	120.0
C20—C19—C18	120.1 (2)	C38—C39—C40	120.2 (2)
C20—C19—H19A	120.0	C38—C39—H39A	119.9
C18—C19—H19A	120.0	C40—C39—H39A	119.9
C19—C20—C15	120.8 (2)	C39—C40—C35	120.7 (2)
C19—C20—H20A	119.6	C39—C40—H40A	119.6
C15—C20—H20A	119.6	C35—C40—H40A	119.6
			
C8—N1—C1—C2	−0.2 (5)	C23—N3—C22—C21	−0.3 (4)
N1—C1—C2—C3	1.1 (5)	C28—C21—C22—N3	−0.5 (5)
C1—C2—C3—C4	−1.0 (4)	C22—N3—C23—C27	0.3 (4)
C1—C2—C3—P1	−178.6 (2)	C22—N3—C23—C24	−179.5 (2)
C9—P1—C3—C2	−1.9 (2)	N3—C23—C24—C25	−179.1 (2)
C15—P1—C3—C2	−106.8 (2)	C27—C23—C24—C25	1.1 (3)
C9—P1—C3—C4	−179.43 (17)	N3—C23—C24—P2	−2.5 (3)
C15—P1—C3—C4	75.65 (18)	C27—C23—C24—P2	177.73 (17)
C5—N2—C4—C8	0.3 (3)	C29—P2—C24—C25	−110.2 (2)
C5—N2—C4—C3	−179.9 (2)	C35—P2—C24—C25	−5.1 (2)
C2—C3—C4—N2	−179.5 (2)	C29—P2—C24—C23	73.42 (19)
P1—C3—C4—N2	−1.7 (3)	C35—P2—C24—C23	178.59 (17)
C2—C3—C4—C8	0.3 (3)	C23—C24—C25—C26	−0.3 (4)
P1—C3—C4—C8	178.06 (17)	P2—C24—C25—C26	−176.7 (2)
C4—N2—C5—C6	−0.8 (4)	C27—N4—C26—C25	1.4 (4)
N2—C5—C6—C7	0.5 (4)	C24—C25—C26—N4	−1.0 (4)
C5—C6—C7—C8	0.3 (4)	C26—N4—C27—C28	178.6 (3)
C1—N1—C8—C7	179.3 (3)	C26—N4—C27—C23	−0.5 (4)
C1—N1—C8—C4	−0.6 (4)	N3—C23—C27—N4	179.5 (2)
C6—C7—C8—N1	179.4 (3)	C24—C23—C27—N4	−0.7 (4)
C6—C7—C8—C4	−0.7 (4)	N3—C23—C27—C28	0.4 (4)
N2—C4—C8—N1	−179.7 (2)	C24—C23—C27—C28	−179.8 (2)
C3—C4—C8—N1	0.6 (4)	C22—C21—C28—C27	1.2 (5)
N2—C4—C8—C7	0.4 (4)	N4—C27—C28—C21	179.7 (3)
C3—C4—C8—C7	−179.3 (2)	C23—C27—C28—C21	−1.2 (4)
C3—P1—C9—C10	−101.0 (2)	C24—P2—C29—C34	10.2 (2)
C15—P1—C9—C10	2.5 (2)	C35—P2—C29—C34	−93.9 (2)
C3—P1—C9—C14	83.5 (2)	C24—P2—C29—C30	−168.96 (18)
C15—P1—C9—C14	−172.9 (2)	C35—P2—C29—C30	86.93 (19)
C14—C9—C10—C11	0.8 (4)	C34—C29—C30—C31	0.3 (4)
P1—C9—C10—C11	−174.7 (2)	P2—C29—C30—C31	179.5 (2)
C9—C10—C11—C12	−1.1 (5)	C29—C30—C31—C32	−0.5 (4)
C10—C11—C12—C13	0.8 (5)	C30—C31—C32—C33	0.4 (4)
C11—C12—C13—C14	−0.3 (6)	C31—C32—C33—C34	−0.1 (4)
C12—C13—C14—C9	0.0 (6)	C30—C29—C34—C33	0.1 (3)
C10—C9—C14—C13	−0.3 (5)	P2—C29—C34—C33	−179.12 (19)
P1—C9—C14—C13	175.5 (3)	C32—C33—C34—C29	−0.1 (4)
C9—P1—C15—C16	106.28 (18)	C24—P2—C35—C36	86.70 (19)
C3—P1—C15—C16	−149.62 (18)	C29—P2—C35—C36	−167.86 (17)
C9—P1—C15—C20	−72.8 (2)	C24—P2—C35—C40	−96.5 (2)
C3—P1—C15—C20	31.4 (2)	C29—P2—C35—C40	8.9 (2)
C20—C15—C16—C17	−1.0 (3)	C40—C35—C36—C37	0.9 (4)
P1—C15—C16—C17	179.93 (19)	P2—C35—C36—C37	177.87 (19)
C15—C16—C17—C18	1.7 (4)	C35—C36—C37—C38	−1.7 (4)
C16—C17—C18—C19	−1.0 (4)	C36—C37—C38—C39	1.1 (4)
C17—C18—C19—C20	−0.3 (4)	C37—C38—C39—C40	0.4 (5)
C18—C19—C20—C15	1.0 (4)	C38—C39—C40—C35	−1.2 (4)
C16—C15—C20—C19	−0.4 (3)	C36—C35—C40—C39	0.5 (4)
P1—C15—C20—C19	178.64 (17)	P2—C35—C40—C39	−176.3 (2)
